# Bright and Stable Nanomaterials for Imaging and Sensing

**DOI:** 10.3390/polym15193935

**Published:** 2023-09-29

**Authors:** José Paulo Sequeira Farinha

**Affiliations:** Centro de Química Estrutural, Institute of Molecular Sciences and Departamento de Engenharia Química, Instituto Superior Técnico, Universidade de Lisboa, 1049-001 Lisboa, Portugal; farinha@tecnico.ulisboa.pt

**Keywords:** bright polymer nanomaterials, fluorescence microscopy imaging, fluorescence sensing, fluorescent polymer chains, fluorescent polymer nanoparticles, fluorescent silica nanoparticles, plasmonic emission enhancement

## Abstract

This review covers strategies to prepare high-performance emissive polymer nanomaterials, combining very high brightness and photostability, to respond to the drive for better imaging quality and lower detection limits in fluorescence imaging and sensing applications. The more common approaches to obtaining high-brightness nanomaterials consist of designing polymer nanomaterials carrying a large number of fluorescent dyes, either by attaching the dyes to individual polymer chains or by encapsulating the dyes in nanoparticles. In both cases, the dyes can be covalently linked to the polymer during polymerization (by using monomers functionalized with fluorescent groups), or they can be incorporated post-synthesis, using polymers with reactive groups, or encapsulating the unmodified dyes. Silica nanoparticles in particular, obtained by the condensation polymerization of silicon alcoxides, provide highly crosslinked environments that protect the dyes from photodegradation and offer excellent chemical modification flexibility. An alternative and less explored strategy is to increase the brightness of each individual dye. This can be achieved by using nanostructures that couple dyes to plasmonic nanoparticles so that the plasmon resonance can act as an electromagnetic field concentrator to increase the dye excitation efficiency and/or interact with the dye to increase its emission quantum yield.

## 1. Introduction

Optical techniques for imaging and diagnosis are at the center of the wonderous developments in the biomedical field, which are paving the way to personalized medicine. Fluorescence-based techniques, in particular, have allowed dramatic progress in the analysis of biological systems and other complex processes. This is because they present better specificity, sensitivity, contrast, and versatility than techniques based on the absorption/reflection of light [1]. Their performance is closely related to the brightness and photostability of the light-emitting materials: the amount of light emitted for a given illumination and the ability to withstand high-power illumination without degrading.

One can distinguish two main types of fluorescence imaging approaches. The first relies on the intrinsic fluorescence of some materials, such as biological tissues, crude oil, plants, etc. The second approach uses fluorescent dyes that can be designed to label specific locations in a sample, providing very high specificity (for example, targeting tumor markers, genes, mitochondria, membranes, or amyloid plaques in Alzheimer’s-associated tissue) [2]. Furthermore, by using fluorophores with sensing capabilities for specific chemical species (pH, glucose, ions, oxygen, biomolecules, etc.) or other parameters (temperature, etc.), it is possible to map their distribution [3].

Fluorescence imaging methods include a variety of techniques in which one measures fluorescence intensity, lifetime, polarization, or the effects of resonance energy transfer, quenching, etc. Although optical imaging is intrinsically limited to resolutions of ca. 300 nm by the diffraction of light, over the last 20 years, a number of methods based on laser-scanning technology have been developed to circumvent this limitation. This is done by detecting the position of only a few fluorescence species at a time and reconstructing their distribution. The main super-resolution optical microscopy techniques, stimulated emission depletion (STED) [4], photo-activated localization (PALM) [5], stochastic optical reconstruction microscopy (STORM) [6], and other variants, all require prolonged and intense illumination of the samples.

To take full advantage of the high resolution and low detection limits of fluorescence imaging and analysis techniques and to respond to the drive for better imaging quality and lower detection limits, it is paramount to develop fluorescent probes and labels that combine very high brightness and photostability. Both the resolution and sensitivity of these techniques depend on the number of photons that are emitted by the fluorescent species. However, there is a tradeoff between using high illumination power to achieve higher resolution or detection and the photodegradation of the dyes (as well as the damage to the samples, including phototoxicity in biological samples). Therefore, to improve resolution and detection limits, as well as to extend the time over which a probe can be observed, one must increase the number of photons emitted by the dyes for a given illumination intensity (i.e., increase the brightness of the dyes) [7] and increase their photostability [8].

These characteristics can be greatly improved by using nanomaterials instead of luminescent proteins and small molecules. Brighter nanomaterials can be obtained by combining many dyes in a single reporter unit, or carrier. The choice of materials for the reporter unit should follow a number of design parameters, in particular: (i) it should avoid contact between the dyes (to avoid emission quenching); (ii) the number of dyes per carrier should be homogeneous (to obtain a uniform response); (iii) the carrier should shield the dyes from oxygen (to avoid photodegradation); (iv) the dyes should not leach from the carrier; and (v) the carriers should be non-toxic if biological applications are envisaged.

Polymer nanomaterials provide the design flexibility to overcome the inherent brightness and photostability issues of fluorescent dyes [9,10,11]. These materials can incorporate large numbers of fluorescent dyes and work as a single reporter unit in either imaging or sensing applications. By confining a large number of dyes in a nanostructure, one can increase the overall absorption coefficient. However, it is only possible to obtain a large increase in the brightness of the nanoreporter if the dyes do not strongly reduce their emission efficiency (fluorescence quantum yield) upon incorporation in the nanomaterial. Unfortunately, such reductions are common because the large dye concentrations necessary to achieve high absorption coefficients in the nanomaterials often result in emission quenching, defeating the purpose of using such high concentrations [12]. Emission quenching occurs due to aggregation of the dyes, caused by the small distances between dyes in the nanostructure and the usually stacking-prone, fairly planar aromatic structure of most fluorescent dyes [13]. To achieve very bright nanostructures, it is therefore paramount to guarantee a homogeneous distribution of the dyes and to prevent interaction between them. Although an increase in the size of the nanostructure would in principle increase the spacing between the dyes and thus reduce quenching effects, the increase in size also increases light scattering, resulting in poor excitation and low emission detection efficiency. In this context, it is important to guarantee the colloidal stability of the nanomaterials to avoid their aggregation into big structures that also cause strong light scattering. Furthermore, many applications of emissive nanomaterials have inherent requirements regarding their size, for example, when these materials have to cross high biological barriers, such as the cell nucleus, the blood-brain barrier, etc. [14].

Strategies to reduce fluorescence quenching usually rely on achieving a random distribution of the dye molecules when these are incorporated in nanomaterials [15,16], modifying the dyes with bulky side groups [17], using bulky counterions [18], or using dyes that assemble into dye nanoparticles with aggregation-induced emission (AIE) [19,20].

Another aspect to consider is the homogeneity of the optical response of the nanostructures. Both in imaging and sensing applications, it is important to have a uniform response per reporter so that each occurrence of a particular feature or event corresponds to a given emission intensity. To achieve this, one must prepare the nanostructures with a uniform number of dyes per reporter [16].

Emission reliability over time is also important to allow long observation/detection times without a drift in signal intensity. The incorporation of fluorescent molecules in nanomaterials usually enhances photostability relative to isolated fluorescent molecules. This is due to two factors. First, many nanostructures usually feature less photodegradation of the organic dyes under illumination due to the lower concentration and/or lower diffusivity of oxygen inside the nanostructure. Second, by having a large number of dyes per structure, the characteristic blinking of individual fluorescent dyes under continuous illumination (which go through off states with no emission, most often due to intersystem crossing to a triplet state and electron transfer) [21] is averaged out.

Finally, for biological applications, one must also consider the biocompatibility of the nanomaterials and their surface chemistry to prevent toxic effects and unspecific interactions that would hinder the labeling of the intended targets [22,23]. The cytotoxicity of nanomaterials depends, of course, not only on their chemical composition but also on their surface chemistry and morphology [24]. Most nanomaterials can be easily internalized into cells and tissues, and their surface chemistry can be tuned to target specific biological sites. Many different types of photoluminescent nanomaterials are described in the literature [11,25], from polymer chains of well-controlled size and architecture containing multiple dyes to dye-loaded polymer nanoparticles (PNPs) that cover the size range from tens to hundreds of nanometers, dye-loaded silica nanoparticles (SNPs) obtained by condensation polymerization of different alcoxisilanes [26,27], semiconductor nanoparticles (quantum dots, QDs), [28] aggregation-induced emission (AIE) fluorescent nanoparticles, [29,30] conjugated polymer nanoparticles (CPNPs) [31], lanthanide-doped upconverting nanoparticles (UCNPs) [32]. Other less common types include silicon quantum dots, [33] dye-loaded dendrimers, [34] lipid nanoparticles [35], etc. [11]. Finally, the more recent additions to this list include emissive gold nanoclusters [36,37] and carbon nanomaterials [38,39]. These nanomaterials have different degrees of performance and flexibility regarding their emission properties and chemistry [9]. For example, in the case of QDs [40,41] and UCNPs [42], the optical properties depend on their size/structure, so their brightness cannot be tuned independently from the emitted color. Their use in biological media further requires encapsulation to reduce the leaching of toxic products and possible long-term toxicity issues. Lanthanide-doped luminescent nanoprobes [43] and AIE nanoparticles [44] usually present relatively low extinction coefficients and excitation wavelengths, mostly in the UV-visible range. AIE nanomaterials have recently been surpassing these shortcomings, with examples featuring absorption and emission extending to the NIR with acceptable brightness and longer-term biocompatibility, targeting theragnostic applications [45]. Recently, emissive carbon nanomaterials [46,47] and gold nanoclusters [37,48,49] have been gaining importance in the field due to their tunable fluorescence and excellent biocompatibility, but these materials are still limited by their relatively low brightness. Gold nanoclusters also often have limited colloidal stability, which can be circumvented by encapsulating them in different materials [37]. Fluorescent nanomaterials based on organic fluorophore-containing polymers, silica, and their hybrids are arguably those offering better control over emission brightness and wavelength, particle size, and surface chemistry [9,50].

A radically different and less explored approach to increasing the brightness of luminescent materials is to increase the light emitted by each individual dye. This can be achieved by coupling the dyes to plasmonic nanoparticles. Such constructs use the plasmon resonance as an electromagnetic field concentrator to increase the excitation efficiency and/or quantum yield of the dyes, enhancing the dye brightness [51,52,53,54]. Coupling dyes to plasmonic nanoparticles is an elegant approach to increasing dye brightness, and although it is a far from widespread use, applications in imaging and sensing have already been proposed [55,56].

**Figure 1:**
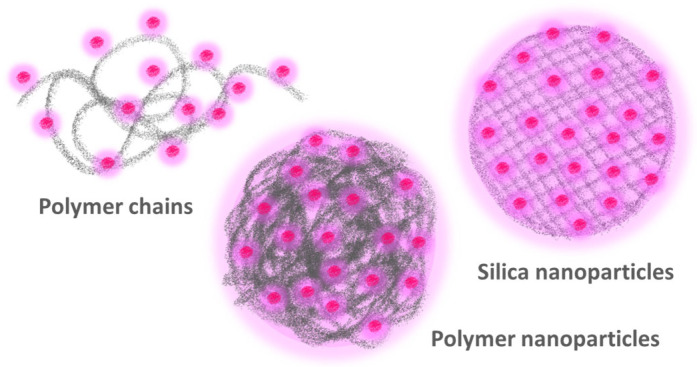
The more commonly used approaches to obtaining highly fluorescent polymer nanomaterials by incorporating a large number of fluorescent dyes are to attach the dyes to individual polymer chains (**left**) and to encapsulate the dyes in polymer (**center**) or silica (**right**) nanoparticles.

This review is focused on the more commonly used highly fluorescent nanomaterials, starting with the two major approaches to designing polymer nanomaterials carrying a large number of fluorescent dyes (Figure 1), namely, to attach them to individual polymer chains (Section 2) and to encapsulate the dyes in polymer nanoparticles (Section 3). In the first approach, the dyes are attached to polymer chains, either during the polymerization reaction or post-synthesis, using previously incorporated reactive monomer groups. The second case is generally limited to diameters above ca. 30 nm and can either rely on the encapsulation of free dyes or on the use of functionalized dyes that are covalently linked to the polymer during polymerization. Silica nanoparticles are then covered as a particular type of highly crosslinked polymer nanoparticles (Section 4), which offer great flexibility and control of size, morphology, and chemical surface modification. Finally, we briefly discuss a bolder and less explored approach based on building nanostructures that couple fluorescent dyes with plasmonic nanoparticles to increase the brightness of each dye (Section 5). Here, control of the structure morphology is paramount because the dye-metal distance must be tuned very precisely to obtain an emission enhancement.

## 2. Polymer Chains with Multiple Fluorescent Dyes

Polymer chains of low molecular weight dispersity, well-defined architecture, and a controlled number of dyes provide extremely well-controlled vehicles that can cover a size range of only a few nanometers (well below the minimum size achievable by polymer nanoparticles). These vehicles are particularly promising owing to their versatility in terms of the monomers and mixtures of monomers that can be used and their modification with different groups (fluorescent, targeting groups, etc.). Polymer chains can be prepared with a very narrow size distribution and precisely controlled architecture and composition by using synthetic approaches based on controlled radical polymerization techniques [57]. To obtain a fluorescent polymer chain reporter with a homogeneous signal per reporter unit, one should covalently attach a large and known number of fluorescent dyes to the polymer chain so that they do not interact with one another (to avoid quenching of the emission).

**Figure 2:**
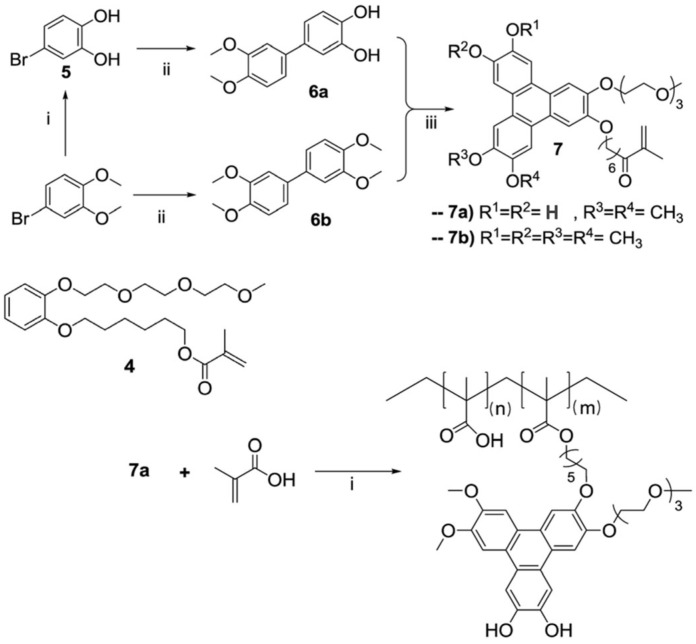
Synthetic route to obtain an asymmetric triphenylene fluorescence boron sensor: (i) BBr_3_, CH_2_Cl_2_, −78 °C to rt, 16 h, quantitative yield; (ii) 3,4-dimethoxyphenyl boronic acid, Pd(PPh_3_)_4_, K_2_CO_3_, toluene, 125 °C, 16 h (**6a**, 41%; **6b,** 69%); (iii) **4**, FeCl_3_, CH_2_Cl_2_ or 1,4-dioxane, 1 h 30 min, rt, reacted with **6a** or **6b** to yield **7a** (25%) or **7b** (23%), respectively. Copolymerization of **7a** with methacrylic acid yielded the polymeric boron sensor **7a-PMAA**: **7a** (1 equivalent), methacrylic acid (20 equivalents), AIBN, DMF, 80 °C, 16 h, quantitative mass yield (Adapted with permission from Ref. [59] Copyright Royal Society of Chemistry).

One approach to incorporating fluorescent dyes or sensor groups in the polymer chains is to modify them with polymerizable groups. For example, a new fluorescent boron sensor, 2,3,6,7,10,11-Hexahydroxytriphenylene [58], was modified with an acrylate group for copolymerization with acrylic acid to increase the signal in boron detection (Figure 2) [59,60,61]. Although this strategy can be quite cumbersome, it can be adapted for different fluorescent dyes [62,63] in order to incorporate them in different polymer chains [64,65,66,67,68,69]. However, the incorporation of dyes by copolymerization often leads to poor homogeneity of the dye distribution along the chain due to the usually large difference in reactivity ratios between monomers containing dyes and other monomers. An inhomogeneous distribution of the dyes along the chain can lead to emission quenching at high dye content.

**Figure 3:**
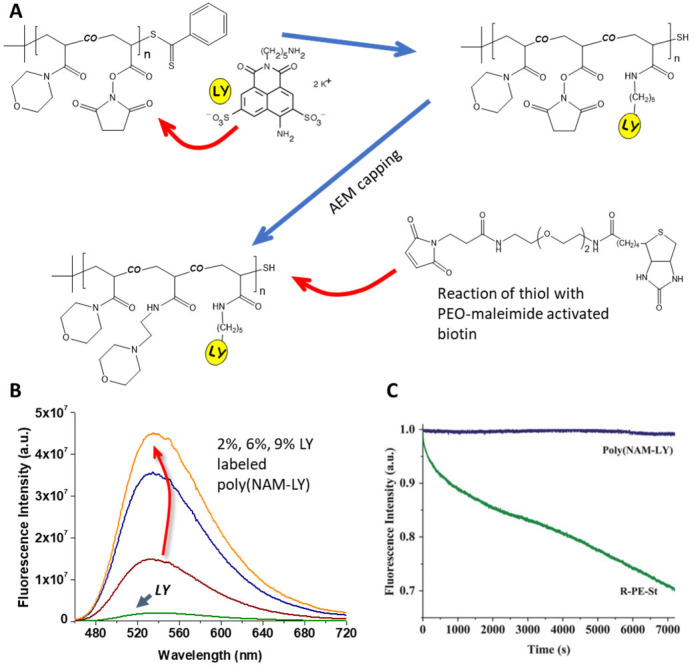
(**A**) Synthetic pathway for the binding of Lucifer yellow cadaverine (LY) to a copolymer of N-acryloylmorpholine (NAM) and the reactive monomer N-acryloxysuccinimide (NAS) obtained by RAFT-controlled polymerization, followed by capping with aminoethylmorpholine (AEM) of the remaining NAS and reaction of the resulting thiol chain-end group with a biotine derivative. (**B**) Fluorescence spectra of aqueous equimolar solutions of LY and of the fluorescent polymer poly(NAM-Ly) labeled with 2, 6, and 9 mol% of LY, showing a 7-, 20-, and 43-fold increase in emission, respectively. (**C**) The poly(NAM-LY) fluorescent polymer also shows excellent resistance to photobleaching, as shown by the fluorescence emission intensity (blue line, monitored at 525 nm), compared to that of R-phycoerythrin-streptavidine (green line, monitored at 578 nm), for 2 h of irradiation at 430 and 490 nm excitation, respectively, in a phosphate buffer (Adapted with permission from Ref. [16] Copyright Royal Society of Chemistry).

Another strategy is to copolymerize a reactive monomer, which can be later used to couple a fluorescent group to the polymer. In this case, the different monomers involved should have very similar reactivity ratios to guarantee a homogeneous distribution of the reactive monomers along the polymer chain [70,71]. This results in a homogeneous dye distribution, which, in the absence of other quenching groups in the polymer [72], allows the development of fluorescent polymer probes with very high brightness. From the few examples described in the literature, one successful example is a bright fluorescent water-soluble reporter based on a statistical polymer of N-acryloylmorpholine (NAM) and N-acryloxysuccinimide (NAS), end-functionalized with biotin (a biomolecule for biotin–avidin click chemistry), prepared by RAFT polymerization (Figure 3) [16]. Using the NAS monomer units, the chains were further labeled with 7 to 62 lucifer yellow (LY) fluorophores per polymer chain, yielding polymers with low fluorescence self-quenching and a 7- to 43-fold increase in brightness compared to free LY [16]. The polymer chains have precisely controlled architecture, very low size dispersity, random dye distribution, good resistance to photobleaching, no sensitivity to pH, and feature a chain-end biotin ligand to promote specific recognition with the streptavidin protein [73,74,75], leading to the development of polymer probes for signal amplification in biosensing assays, labeling of cell biotin receptors in cancer studies, labeling of antibodies, and microscopy imaging [76,77,78].

## 3. Polymer Nanoparticles

An alternative approach is to use polymer nanoparticles (PNPs) to incorporate the dyes. Although nanoparticles from conjugated polymers are intrinsically fluorescent and very bright [79,80,81,82], they have limited application because of poor biodegradability and cumbersome stabilization. On the other hand, nanoparticles of non-fluorescent polymers or copolymers can be used to encapsulate fluorescent dyes, offering huge versatility due to the large number of available monomers, the possibility to mix different monomers, and the incorporation of different groups in the polymer. Additionally, there are a large number of different preparation procedures, and the polymers can be obtained in a wide range of sizes. The incorporation of fluorescent dyes in these nanoparticles can rely solely on the encapsulation of the molecules (a simple approach that can lead to the leaching of the dyes from the nanoparticles) or on the modification of monomers with fluorescent groups (which prevents dye leaching but involves more complex synthetic procedures).

**Figure 4:**
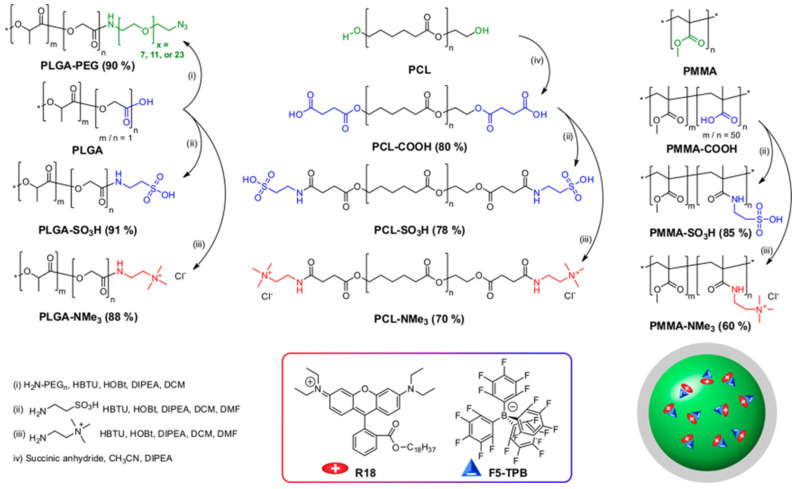
Synthesis of dye-loaded NPs by charge-controlled nanoprecipitation of poly(d,l-lactide-co-glycolide) (PLGA) with a carboxylate group at one chain end, polycaprolactone (PCL) with hydroxyl groups at both chain ends, and a copolymer of poly(methyl methacrylate) (PMMA) with methacrylic acid were modified with PEG, carboxylate, sulfonate, and trimethylammonium groups. A salt of rhodamine B octadecyl ester (R18) with tetrakis(pentafluorophenyl)borate (F5-TPB) as a counterion was used as dye for encapsulation (Adapted with permission from Ref. [85] Copyright American Chemical Society).

Dye-loaded polymer nanoparticles can be obtained from preformed polymer chains by a number of different approaches, but these are usually associated with poor control over the size of the nanoparticles, their stability, and the distribution of the dyes in the nanoparticles. Emulsification of preformed polymers involves previously dissolving the polymer (and the dyes) in a water-immiscible, low boiling point solvent that is dispersed in water using a surfactant and strong shearing (mechanical or ultrasound) to yield nanoparticles with diameters in the hundreds of nanometers range [83]. In nanoprecipitation, the polymer with the dyes is dissolved in a water-miscible solvent, which, when added to water, leads to the (kinetically controlled) formation of nanoparticles in a wide range of diameters [84]. In one example, charge-controlled nanoprecipitation was proposed to improve the morphology control of the nanoparticles and the dye encapsulation using charged polymers and a salt of rhodamine B octadecyl ester (R18) with tetrakis(pentafluorophenyl)borate (F5-TPB) as a counterion (Figure 4) [85].

Self-assembly of amphiphilic copolymers can also lead to nanoparticles (copolymer micelles), but since the process is generally thermodynamically driven, the nanoparticle stability can be drastically decreased at low concentrations, and the system must be carefully considered for different media [86]. The three methods can be adapted to use polymers previously labeled with covalently attached fluorescent dyes; however, since they rely on differences in solvation, the impact of the presence of the dyes in the polymer must be evaluated on an individual basis [87].

An approach offering better control over particle morphology is to obtain the nanoparticles directly by polymerizing the monomers. There are three major techniques for this, which involve different particle formation mechanisms: emulsion, miniemulsion, and microemulsion polymerization.

**Figure 5:**
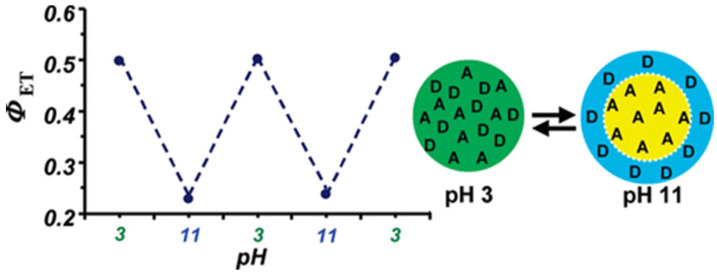
FRET two-component polymer nanoparticles undergo reversible morphology transformation in water as a function of pH. The particles consist of a high molecular weight acrylate copolymer and an acid-rich oligomer designed to be miscible with the polymer when its carboxylic groups are protonated. Attaching a pair of fluorescence resonance energy transfer (FRET) dyes to the components inside the nanoparticles enabled the determination of the particle morphology at the molecular level, showing that the components are miscible in acidic conditions (higher FRET efficiency, *ϕ*_ET_) but undergo charge-induced phase separation (lower *ϕ*_ET_) when the oligomers are neutralized to their carboxylate form (Adapted with permission from Ref. [93] Copyright American Chemical Society).

In the preparation of fluorescent polymer nanoparticles by emulsion polymerization, the monomers are emulsified in water, usually with one or more surfactants, with the initial system containing micron-size monomer droplets and surfactant micelles. The polymerization starts in the surfactant micelles due to their larger overall surface area, and the droplets act as monomer reservoirs to feed the particle growth in the micelles. Due to this mechanism, emulsion polymerization is very efficient in producing nanoparticles with extremely narrow size distributions and diameters ranging from ca. 30 nm to hundreds of nanometers. However, dye encapsulation is difficult because hydrophobic dyes (low solubility in water) tend to stay in the monomer droplets instead of migrating to the micelles, from where the particles grow. On the other hand, water-soluble dyes stay in the water phase, also leading to poor incorporation. This limitation can be circumvented by using fluorescent dyes modified with polymerizable groups [88,89] and incorporating them into the polymer chains using a starved feed approach where the monomer mixture is fed to the reactor at a controlled rate [90,91]. This allows the preparation of fluorescent nanoparticles with very homogeneous dye distribution and no aggregation or dye leaching for demanding applications requiring precise control of the nanoparticle size, morphology, and optical properties [92]. The disadvantages of this approach to producing bright fluorescent nanoparticles are that the particle size is relatively large (leading to light scattering when in dispersion), and it is difficult to reach very high dye content. Nevertheless, this approach has been extremely successful in preparing PNPs for characterizing polymer interphases by Forster resonance energy transfer (FRET), for example in waterborne polymer coatings prepared from mixtures of PNPs labeled with donor and acceptor dyes [92]. In one example, two-component polymer nanoparticles prepared with a high molecular weight acrylate copolymer and an acid-rich oligomer, each labeled with a fluorescence dye that forms a FRET pair, were shown to be homogeneous in acidic conditions (higher FRET efficiency between the dyes, *ϕ*_ET_), but to undergo charge-induced reversible phase separation when the oligomers are neutralized to their carboxylate form. (Figure 5) [93].

**Figure 6:**
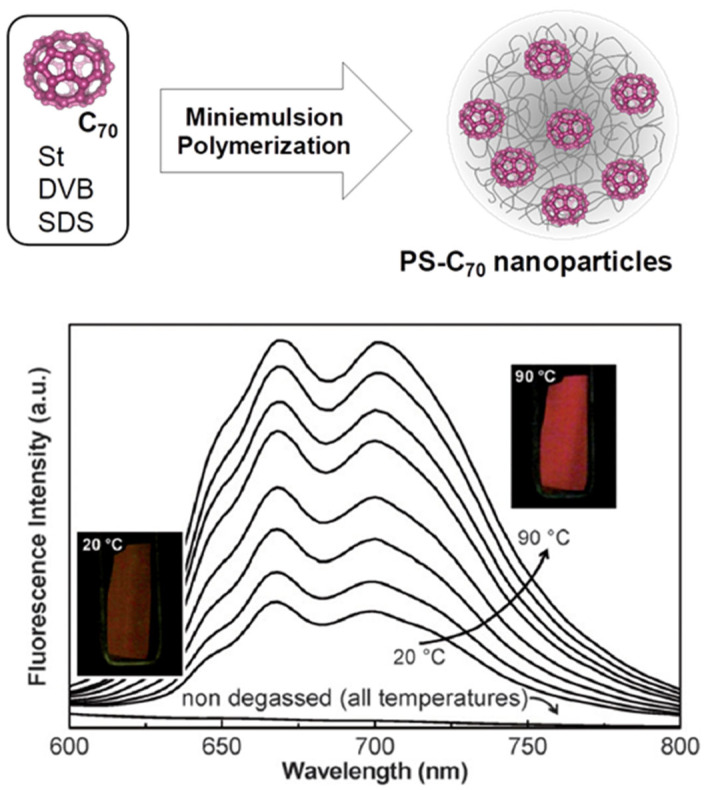
Encapsulation of fullerene C70 in polystyrene nanoparticles (PS) by miniemulsion polymerization (**top**). The fluorescence emission spectra of a degassed film of PS-C70 nanoparticles (bottom) show the strong temperature dependence of the thermally activated delayed fluorescence (TADF) of C70. The photos show the film under UV illumination at 20 °C and 90 °C (Adapted with permission from Ref. [96] Copyright Royal Society of Chemistry).

Miniemulsion polymerization offers lower control over particle size than emulsion polymerization but is much better suited for the encapsulation of hydrophobic molecules such as fluorescent dyes. The nanoparticles obtained by miniemulsion polymerization can be smaller than those obtained by emulsion polymerization (ca. 30 to 200 nm). In miniemulsion polymerizations, the droplet size is reduced by applying high shear forces (mechanical stirring or sonication), with the droplets stabilized by a surfactant and a water-insoluble co-stabilizer (e.g., hexadecane) [94]. The polymerization occurs inside the monomer droplets, which directly originate the nanoparticles. Therefore, dyes or other hydrophobic molecules initially present in the monomer droplets become encapsulated in the resulting nanoparticles. Miniemulsion polymerization is especially useful for the encapsulation of dyes that cannot be functionalized without affecting their optical properties, as in the case of fullerenes. In one example, C70 was successfully incorporated into polystyrene nanoparticles (PS) by miniemulsion polymerization so that the nanoparticles could be incorporated into different materials where C70 is not dispersible. Moreover, the strong temperature dependence of the thermally activated delayed fluorescence (TADF) of C70 was maintained in the PS-C70 nanoparticles (Figure 6) [95,96]. However, monomer-modified dyes have also been encapsulated by copolymerization in miniemulsions [97].

Microemulsion polymerization relies on thermodynamically stable emulsions of very small droplets stabilized with high amounts of surfactants [98], allowing the preparation of very small nanoparticles (from ca. 5 nm). The use of thermodynamically stable emulsions strongly limits the diversity of monomers that can be used in microemulsion and the application of this technique. One other disadvantage of microemulsion polymerization for preparing dye-loaded nanoparticles is that the dye distribution is not homogeneous among particles due to inhomogeneous nucleation (occurring in both droplets and in micelles). In this approach, the dyes can also be copolymerized [99] or incorporated after particle formation by swelling the nanoparticles [100].

**Figure 7:**
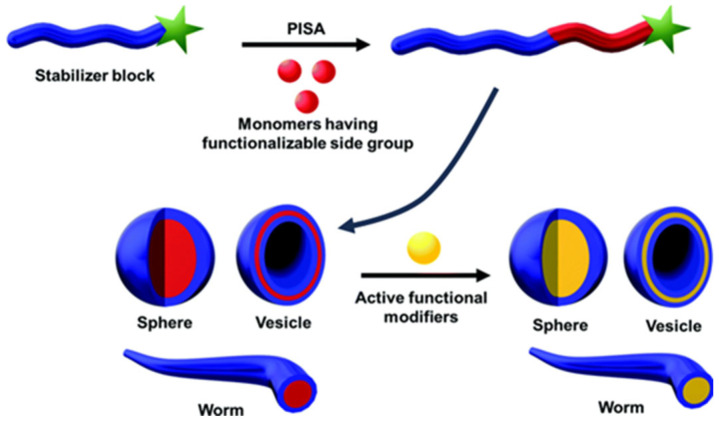
Nanostructures produced by polymerization-induced self-assembling (PISA). The structures are self-assembled in situ during the formation of a diblock copolymer with a core-forming block and an outer block providing colloidal stability, followed by post-functionalization with an active functional modifier (adapted with permission from Ref. [102] Copyright Royal Society of Chemistry).

A more recent technique to obtain nanoparticles directly by polymerizing the monomers is [101]. In this case, the growing hydrophobic block of amphiphilic copolymer chains induces the self-assembling of the polymer chains into nanoparticles (Figure 7). Incorporation of fluorescent dyes can rely on the encapsulation in the hydrophobic core or the copolymerization of fluorescent monomers during chain growth [102].

**Figure 8:**
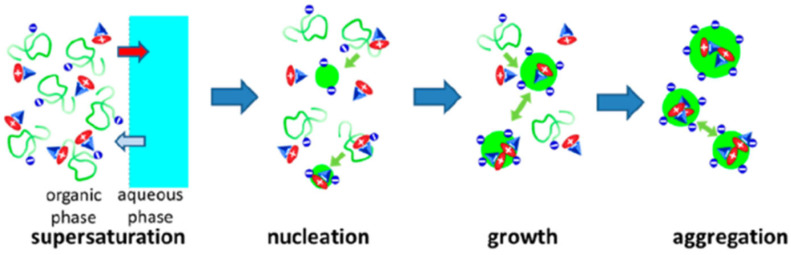
Nanoprecipitation of polymers and dye salt to prepare dye-loaded PNPs. Upon adding the organic phase containing the polymer and the dyes to the aqueous phase, interdiffusion leads to supersaturation of the polymer and/or the dye salt, triggering particle nucleation, growth, and aggregation (Adapted with permission from Ref. [104] Copyright American Chemical Society).

In any of these approaches, to achieve high dye loads in polymer nanoparticles, it is important to control dye aggregation to avoid fluorescence quenching by using starved-feeding techniques (in emulsion polymerization) [90,96,103] or by controlling the nanoprecipitation process to incorporate dyes with bulky side groups [17] or charged dyes with bulky counterions [18]. In the case of nanoprecipitation, the global hydrophobicity of the polymers and the presence of charged groups had a major influence not only on the photophysics of the dyes but also on the size of the PNPs over a range from ∼10 to 200 nm (Figure 8) [104].

## 4. Silica Nanoparticles

Silica is a highly cross-linked polymer obtained by the condensation polymerization of silicon-containing monomers, usually alkoxysilanes, which are hydrolyzed during the condensation process. Silica nanoparticles (SNPs) are widely used in different imaging applications, from optical techniques to magnetic resonance, positron emission, X-ray tomography, and ultrasound, as well as combinations of different techniques [105,106]. SNPs can be prepared by simple, scalable, and low-cost techniques and offer tunable and very well-defined size, morphology, and porosity. One of the most commonly used preparation methods was developed by Stobër, consisting of the base-catalyzed hydrolysis of the silica precursors, followed by condensation polymerization [107]. Precursors such as tetraethyl orthosilicate (TEOS) and other molecules containing alkoxysilane groups are used.

**Figure 9:**
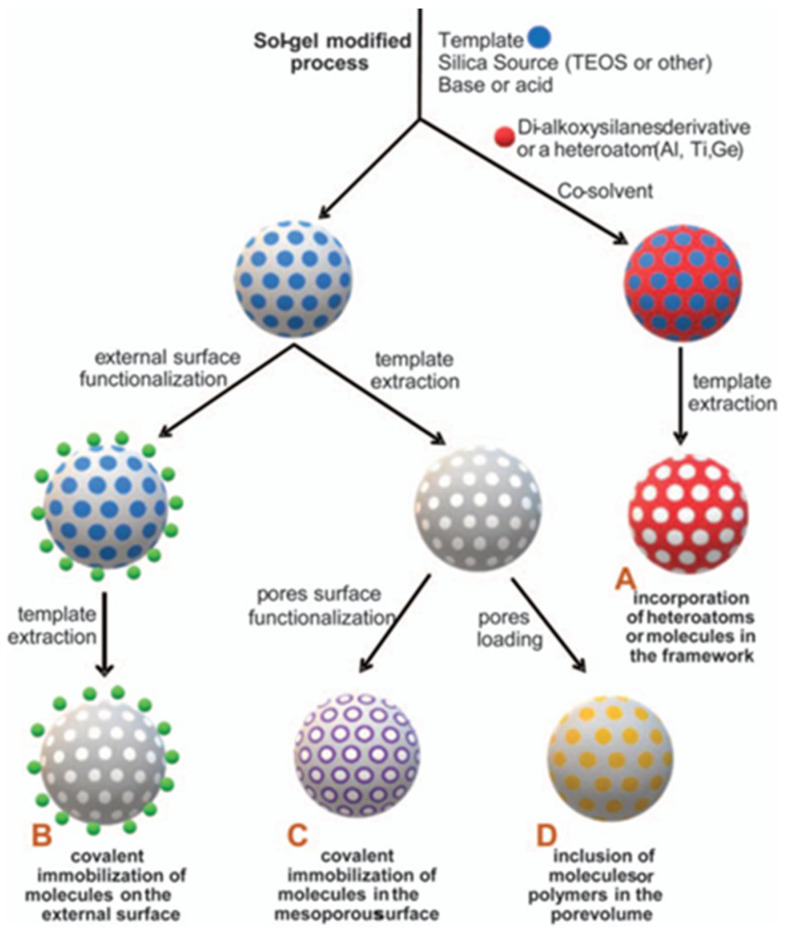
Functionalization of mesoporous silica nanoparticles (MSNs), inside and out. These nanoparticles offer huge chemical modification versatility, with the possibility of combining strategies A to D in a single nanoreporter (Adapted with permission from Ref. [115] Copyright Royal Society of Chemistry).

SNPs feature high chemical and mechanical stability, with the ability to protect guest molecules that are incorporated in the silica structure [108] or entrapped within [109]. Contrary to most PNPs, both the structure and the surface of SNPs can be easily functionalized during and post-synthesis, by well-established siloxane chemistry using organic alkoxysilane compounds for covalent immobilization of different groups. This strategy can be used to improve colloidal stability in different media or to incorporate polymers or (bio)molecules for biological targeting, to modulate interactions, for sensing, to control cargo release, etc. (Figure 9) [108,110,111,112,113,114].

Silica is endogenous to most living organisms, and SNPs are mostly biocompatible, having been long approved for human clinical trials [116]. For biomedical applications, SiNPs of spherical or near-spherical shape and diameters around or under 100 nm are preferred because they are easily internalized in cells, virtually nontoxic, and easily excreted from living organisms. However, in biological environments, bare SNPs are readily coated with proteins and attacked by the immune system, which can impact their performance. To avoid this, they can be surface-modified with polymers or biomolecules that provide stealth and adhesion control properties, with poly(ethylene oxide) being the most common option [117], although other strategies produce even more promising results [118].

SNPs offer excellent support for optical imaging applications since they are transparent in a wide range of wavelengths, from the ultraviolet to the near infrared (NIR) and can incorporate a large number of fluorophores. Fluorescent SNPs can be prepared with very well-defined particle morphology, with diameters ranging from ca. 10 nm to several hundred nanometers [15,119]. They can be doped with fluorophores, either by physically entrapping the dyes inside the particles, by coating the nanoparticle surface, or by covalently attaching the dyes modified with alkoxysilane groups to the silica network [15,27,110,113,120,121]. In all cases, attention has to be paid to the possible aggregation of the dyes that can lead to self-quenching [12,122].

An important advantage of incorporating dyes in SNPs is that the low oxygen diffusivity inside SNPs shields the dyes from oxygen, thus enhancing their photostability (this is especially relevant when using high-power excitation, for example, in laser scanning techniques) [15,123]. Additionally, dye-containing SNPs can be used in solvents where the free dyes are insoluble. Bare dye-loaded fluorescent SNPs are readily dispersed in water, allowing the use of water-insoluble dyes for imaging applications in aqueous media. For use in other environments, SNP can be easily surface modified with appropriate groups.

**Figure 10:**
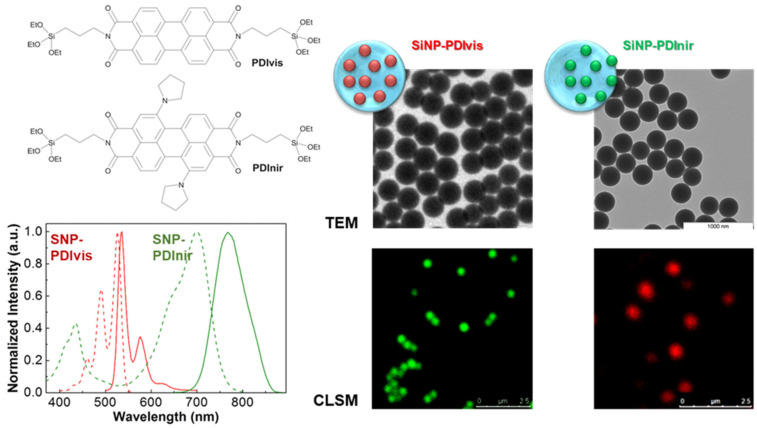
Perylenediimide (PDI) derivatives for anchoring to the silica structure (top left), one with emission in the visible region of the spectrum (PDIvis) and the other emitting in the NIR (PDInir), with the corresponding normalized absorption spectra (dashed lines) and fluorescence spectra (solid lines) measured in 1,4-dioxane (**bottom left**). TEM and laser scanning confocal fluorescence microscopy (LSCFM) images (pseudo color) of silica nanoparticles labeled with PDIvis and SiNP-PDInir (**right**), cast onto a glass slide. The dyes were homogeneously incorporated into monodisperse silica nanoparticles with diameters of ca. 30 to 300 nm, yielding very brightly fluorescent silica nanoparticles, later surface-modified with arginine-glycine-aspartic acid (RGD) tumor-targeting oligopeptides [113,120].

Loading fluorescent dyes into the pores of silica nanoparticles or adsorbing them to the outer particle surface are far easier approaches than incorporating alcoxisilane-modified dyes into the silica structure. However, only the later strategy can effectively avoid the leaching of the dyes from the nanoparticles and the possible contamination of the samples with (often toxic) free dye molecules. The best strategy to prepare luminescent SNPs is thus to covalently link the dyes to the silica structure, not only because this avoids leaching of the dye molecules but also because it offers increased dye chemical and photochemical stability, as well as protection from enzymatic degradation in biological media. For example, the incorporation of different perylenediimide (PDI) derivatives by anchoring these dyes to the silica structure results in nanoreporters with excellent photostability and brightness. Laser scanning confocal fluorescence microscopy (LSCFM) images of the fluorescent silica nanoparticles show a uniform distribution of the dyes among the nanoparticles, with dimensions coinciding with those obtained by TEM, with tunable diameters from ca. 30 to 300 nm, and surface-decorated with tumor-targeting oligopeptides (Figure 10) [124].

## 5. Emission Enhancement Using Plasmonic Nanoparticles

An alternative to producing vehicles with multiple dyes is to increase the amount of light emitted by each dye. This can be achieved by exploring the interaction between the dye exciton and the surface plasmon resonance (SPR) of noble metal nanoparticles. Gold nanoparticles (GNPs) are the most attractive for this application, as their SPR can be tuned in the visible/NIR region of the spectrum (by changing their shape), and they have good chemical stability and biocompatibility, while their surface can be easily modified. GNP-dye constructs can show enhanced brightness (also known as metal enhanced fluorescence, MEF) either due to an increase in the dye excitation efficiency, an increase in its emission quantum yield, or both [52,125,126]. The first phenomenon is related to the increased excitation rate of the dye due to the larger intensity of the local electromagnetic field at the metal surface. In the second case, electronic coupling of the dye and the metal nanoparticle leads to non-radiative transfer of excitation energy to the dye and transmission of the dye energy, as radiation, to the far field by the metal [127]. The two effects depend both on the dye-metal distance and on the spectral overlap of the metal SPR with the emission and excitation of the dye.

To obtain an enhancement of the dye emission, the distance between the dye and the metal nanoparticle should be as small as possible but larger than ca. 5 nm. This distance dependence results from the interplay of two opposite effects. On one hand, quenching of the dye emission by the metal occurs up to 5 nm from the metal surface [128,129] with the dye quantum yield being mostly suppressed for dye-metal distances below 5 nm, as shown by simulation results with a polarizable continuum coupled quantum mechanical model [130]. On the other hand, the electrical field intensity decays exponentially from the metal surface outward, with the enhancement decreasing as the dye-metal separation distance increases [51,131,132,133,134].

Dye-metal nanostructures with increased brightness thus require the use of spacer materials to control the metal-dye distance with precision. This has been attempted using silica [51,131,133,135,136], DNA [137], and polymers [131,138,139,140]. While DNA and polymer spacers can be too flexible to completely prevent dye-metal contact (leading to emission quenching), this problem can be minimized by using very high-density polymer brushes.

**Figure 11:**
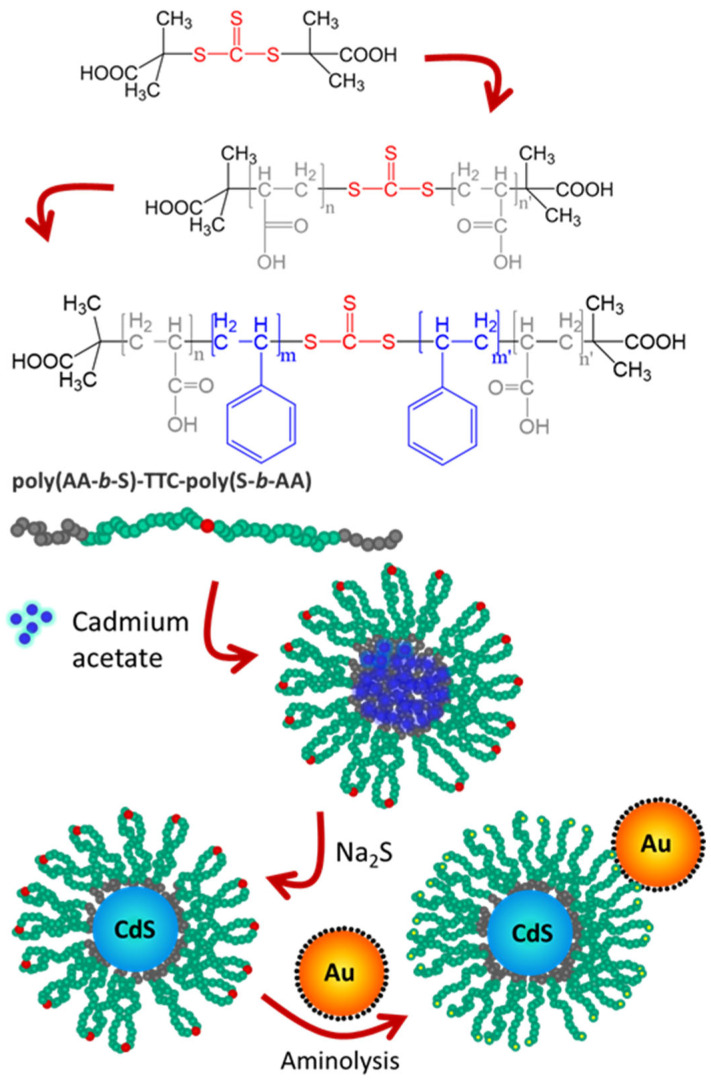
Emission enhancement in constructs of quantum dot micelles (QDM) of Cadmium Sulfide (CdS) and gold nanoparticles (GNP, in yellow). **Top** to **bottom**: RAFT polymerization of a 4-block copolymer, starting from the S,S′-bis(α,α′-dimethyl-α″-acetic acid) trithiocarbonate (TTC) chain transfer agent, grows first the external poly(acrylic acid) blocks (PAA), followed by polymerization of the internal polystyrene blocks (PS). The resulting copolymer (PAA-b-PS-TTC-PS-b-PAA) self-assembles in 1,4dioxane by adding cadmium acetate, forming micelle nanoreactors. CdS quantum dots are then obtained by adding Na_2_S. The micelle shell TTC groups (red dots) are converted into thiol groups (yellow dots) by adding hexylamine and used to anchor the GNPs. (Adapted with permission from Ref. [131] Copyright American Chemical Society).

In one example, a block-copolymer micelle was used as a nanoreactor to grow a single quantum dot (QD) in the core, later attaching the GNPs to the corona of the micelle. This resulted in significant brightness enhancements for different QDs (Figure 11) [131,138].

**Figure 12:**
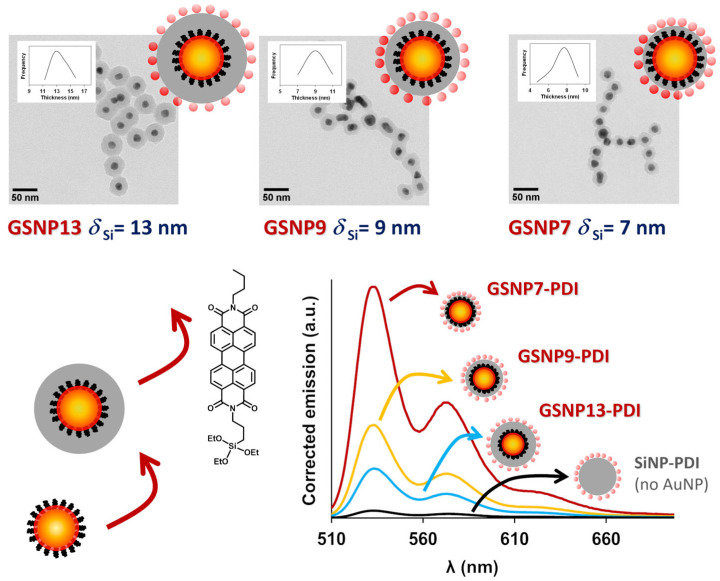
Hybrid core–shell gold–silica nanoparticles (GSNP) with PDI fluorescent dyes attached to the silica surface at a precisely controlled distance from the gold core (7, 9, and 13 nm) were prepared from PEO-modified gold nanoparticles, coated with a silica shell, and decorated with a high quantum yield triethoxysilyl-modified perylenediimide dye (PDI) on the surface (**bottom left**). TEM images of the core–shell structures (**top**) show that the gold core is completely encapsulated in silica (the insets show the silica thickness distribution curves). The corrected emission spectra (**bottom right**) of SNP-PDI (black curve, no gold) and the GSNOs show the increase in dye emission as the dye-metal distance decreases (Adapted with permission from Ref. [51] Copyright Royal Society of Chemistry).

In another example, low-porosity silica was used as a rigid spacer to completely prevent contact between dyes and GNPs, offering excellent control over the dye-metal distance [51]. Other advantages of using silica to encapsulate the GNPs are its robustness, chemical stability, and versatility of surface modification (for example, for the conjugation of biomolecules or dyes [141]). To control the dye-metal distance, a hybrid system was developed with a GNP core and a silica shell of precise thickness. A perylenediimide (PDI) dye derivative with absorption and emission spectra overlapping the SPR of the GNPs and a very high fluorescence quantum yield was covalently attached to the silica outer surface. With this system, it was possible to precisely calculate the emission enhancement for different well-defined dye-metal constructs in dispersion (Figure 12) [51].

Contradictory results on metal-enhanced emission abound in the literature, with metal-dye spacers as large as 90 nm described to produce emission enhancement (and other absurd effects). The reason for such inconsistency on the length scale associated with emission enhancement is probably related to experimental artifacts, such as light scattering and inner filter effects due to the metal nanoparticles. The real enhancement effect in the dye brightness can be obtained by correcting these artifacts [51,131].

Enhancing the brightness of individual reporters by coupling dyes to plasmonic nanoparticles can be extremely relevant to increasing sensitivity in sensing applications [142,143,144] or to being able to use dyes with inherent low brightness while offering other desirable characteristics. This is the case of gold nanoclusters (AuNC), with a diameter under 2 nm and size-dependent emission in the visible/NIR region. These are extremely photostable and biocompatible (depending on the stabilization ligands used), but they have poor colloidal stability and usually low brightness [37]. Co-encapsulation of AuNC and plasmonic nanoparticles in nanomaterials can enhance the AuNC brightness from both the AuNC–AuNP coupling and the large number of AuNC per reporter, as well as the colloidal and photo stability and targeting possibilities imparted by the nanomaterial carrier [145,146].

## 6. Conclusions and Future Perspectives

Fluorescent nanomaterials play a crucial role in the performance of optical microscopy imaging and diagnostic applications because they often determine the contrast/selectivity of imaging and the limits of detection in diagnostic methods. With more demanding applications, the quest for luminescent materials with higher brightness and better photostability led to the development of polymer nanomaterials that can greatly outperform fluorescent proteins and small molecules. The most common approach to developing luminescent polymer nanostructures consists of combining a large number of dyes in a single reporter unit. This strategy has proved very effective in cases where there was no interaction between the dyes (to avoid emission quenching, which lowers emission intensity), a constant number of dyes was loaded per reporter (to guarantee a uniform response between the reporters), and the nanomaterial architecture offered effective shielding from oxygen (to avoid dye photodegradation).

From the different aspects that can be addressed to broaden the super-bight polymer nanomaterials library, the size of the nanoreporters plays an important role in many applications, from cargo delivery across the blood–brain barrier to imaging the cell nucleus, etc. While both polymer chains and polymer/silica nanoparticles can be prepared with precise size and low size dispersion, polymer chain-based carriers provide extremely well-controlled vehicles of only a few nanometers, while polymer/silica nanoparticles cover the size range from a few tens to hundreds of nanometers. There is therefore a gap for diameters around ten nanometers, a region important for applications in the biomedical field. Obtaining well-controlled cargo carriers of such dimensions is still a challenge, requiring the downsizing of current techniques to produce polymer and silica nanoparticles. Polymerization-induced self-assembling (PISA), for example, is an emerging technique that can contribute to filling the 10 nm gap.

The strategy of increasing the brightness of individual reporters by coupling dyes to plasmonic nanoparticles, while involving relatively complex systems (namely, to avoid dye-metal contact leading to very efficient emission quenching), can be extremely relevant for sensing applications and for exploring dyes with inherent low brightness but presenting other desirable characteristics, as for example, gold nanoclusters.

There are still many challenges to overcome for super-bright polymer nanomaterials at the level of fundamental research and some way to go for the development of solutions that can be used on a large scale. However, the huge advantages of these materials are creating a strong need for widespread application in different fields, from super-resolution and 3D optical microscopy to fluorescence-guided surgery, etc.
